# Biological conversion assay using *Clostridium phytofermentans *to estimate plant feedstock quality

**DOI:** 10.1186/1754-6834-5-5

**Published:** 2012-02-08

**Authors:** Scott J Lee, Thomas A Warnick, Sivakumar Pattathil, Jesús G Alvelo-Maurosa, Michelle J Serapiglia, Heather McCormick, Virginia Brown, Naomi F Young, Danny J Schnell, Lawrence B Smart, Michael G Hahn, Jeffrey F Pedersen, Susan B Leschine, Samuel P Hazen

**Affiliations:** 1 Biology Department, University of Massachusetts, Amherst, MA, USA; 2 Plant Biology Graduate Program, University of Massachusetts, Amherst, MA, USA; 3 Department of Microbiology, University of Massachusetts, Amherst, MA, USA; 4 Department of Biochemistry and Molecular Biology, University of Massachusetts, Amherst, MA, USA; 5 BioEnergy Science Center, Complex Carbohydrate Research Center, University of Georgia, Athens, GA, USA; 6 Department of Horticulture, Cornell University, Geneva, NY, USA; 7 USDA-ARS, Grain, Forage, and Bioenergy Research, University of Nebraska-Lincoln, Lincoln, NE, USA

## Abstract

**Background:**

There is currently considerable interest in developing renewable sources of energy. One strategy is the biological conversion of plant biomass to liquid transportation fuel. Several technical hurdles impinge upon the economic feasibility of this strategy, including the development of energy crops amenable to facile deconstruction. Reliable assays to characterize feedstock quality are needed to measure the effects of pre-treatment and processing and of the plant and microbial genetic diversity that influence bioconversion efficiency.

**Results:**

We used the anaerobic bacterium *Clostridium phytofermentans *to develop a robust assay for biomass digestibility and conversion to biofuels. The assay utilizes the ability of the microbe to convert biomass directly into ethanol with little or no pre-treatment. Plant samples were added to an anaerobic minimal medium and inoculated with *C. phytofermentans*, incubated for 3 days, after which the culture supernatant was analyzed for ethanol concentration. The assay detected significant differences in the supernatant ethanol from wild-type sorghum compared with *brown midrib *sorghum mutants previously shown to be highly digestible. Compositional analysis of the biomass before and after inoculation suggested that differences in xylan metabolism were partly responsible for the differences in ethanol yields. Additionally, we characterized the natural genetic variation for conversion efficiency in *Brachypodium distachyon *and shrub willow (*Salix *spp.).

**Conclusion:**

Our results agree with those from previous studies of lignin mutants using enzymatic saccharification-based approaches. However, the use of *C. phytofermentans *takes into consideration specific organismal interactions, which will be crucial for simultaneous saccharification fermentation or consolidated bioprocessing. The ability to detect such phenotypic variation facilitates the genetic analysis of mechanisms underlying plant feedstock quality.

## Background

Lignocellulosic plant biomass is comprised mostly of cell walls, which are a complex composite of proteins, lignin, and polysaccharides; the latter holds promise as raw material for biofuel production. The most abundant polysaccharide in the majority of tissues is cellulose, which exists as unbranched chains containing up to 15,000 β-(1,4)-linked glucose molecules [1]. By contrast, the shorter hemicelluloses are chemically and physically more complex [2]. The most abundant forms exist as glucan chains much shorter than cellulose or β-(1,4)-linked xylose, both with diverse side-chain substitutions of arabinose, galactose, fucose, xylose, or glucuronic acid. Biological conversion relies on an organism, such as a unicellular fungus or bacterium, which will convert these simple sugars to high-energy chemicals such as ethanol or butanol. Unlike seed starch or the soluble sugars found in phloem sap, the fermentable sugars found in cell walls are recalcitrant to extraction. The composition and interaction between the polysaccharides and lignin strongly influence their amenability for conversion to renewable fuels. Whereas lignification has extensive merits for the plant, it has adverse effects on the digestibility by ruminant and biofuel-generating microbes. For example, up to 50% of the variation in *in vitro *digestibility of commercial maize hybrids can be attributed to differences in their lignin content [3]. Lignin is composed ofthree monolignols: *p*-coumaryl, coniferyl, and sinapyl alcohols, which polymerize to form *p*-hydroxyphenyl, guaiacyl, and syringyl phenylpropanoid units, respectively [4]. The biosynthesis of alcohol monomers occurs in a specialized branch of phenylpropanoid metabolism, through which successive reductions, hydroxylations and methylations can occur. Crosslinking lignin with polysaccharides in the secondary cell walls of vascular tissue increases hydrophobicity, and thus gives these functional tissues the capacity to efficiently conduct water [4]. Concurrently, the polysaccharides are less accessible to enzymatic digestion or mechanical penetration by potential pathogens [5]. The pathway for lignin biosynthesis is well conserved among vascular plants, and involves at least 10 gene families, including *CAD *(cinnamyl alcohol dehydrogenase) and *COMT *[6]. Each step in the lignin pathway has been perturbed in various species, resulting in changes in lignin content, composition, and, in many cases, digestibility [7,8].

Genetic diversity of plant cell-wall properties within species is evident in the decades of plant breeding for improved feed and forage quality in crops such as maize, sorghum, and alfalfa [9,10]. The merits of animal feed have been tested frequently *in vivo*, either by evaluating animal performance in response to a particular feeding regimen, or by estimating digestibility *in vivo *using livestock with fistulae [11]. With the latter approach, the gastrointestinal tract of a surgically prepared animal is sampled to measure the remaining biomass. An equivalent *in vitro *method was developed using rumen fluid inoculum from fistulated cows [12]. Digestibility is estimated through analysis of the organic matter lost from the simulated ruminant gut conditions after 4 days of incubation. The throughput of this approach is considerably higher than *in vivo*, and begins to meet the needs of traditional plant-breeding efforts and genetics research. High-throughput assays to estimate feed and forage quality parameters also include compositional measurements of cellulose [13], total lignin and monomer content [14,15], and hemicellulose content and composition [16,17], which also serve as valid measurements of biofuel feedstock quality. Although the parallels between digestibility and amenability to conversion to biofuels might be apparent, industry standards for biofuel feedstock quality are still needed.

Regardless of species, all new crop varieties must meet certain standards for industrial-processing efficiency and consumer-market quality. Beyond the expectation of high biomass yield with few inputs on marginal land, conversion quality standards for energy crops have yet to be defined by the biofuels industry. Recently, several methods, including some high-throughout platforms, have been established that treat plant samples with hydrolytic enzyme cocktails, such as fungal cellulase and xylanase/xylosidase, then assay for total sugars as a measurement of digestibility [18-22]. This approach can be taken one step further, using translational assays that mimic industrial simultaneous saccharification and fermentation (SSF) paradigms, in which the liberated sugars from the polymers can then be fermented by *Saccharomyces cerevisiae *to measure total ethanol yield [23,24].

A distinct and promising approach to cellulosic biofuel production is consolidated bioprocessing (CBP) technology for conversion of biomass to fuel. CBP could lead to a significant reduction in processing costs, greater than the reductions gained from any other potential improvement, such as reducing enzyme loading, eliminating pre-treatment, or improving the processes associated with converting sugars to ethanol [25]. The recently discovered anaerobic forest soil bacterium *Clostridium phytofermentans *may further enhance the efficiency of CBP. This organism produces ethanol as its major fermentation byproduct during growth on all substrates tested, including cellulose, hemicellulose, pectin, and starch [26], as well as switchgrass, corn stover, and pulp wastes ([Warnick and Leschine, unpublished data). Unlike *S. cerevisiae*, of which only engineered strains are capable of limited pentose utilization [27],*C. phytofermentans *directly converts a wide array of fermentable components of biomass to ethanol, including cellulose, pectin, polygalacturonic acid, starch, xylan, arabinose, cellobiose, fructose, galactose, gentiobiose, glucose, lactose, maltose, mannose, ribose, and xylose [26]. Without the addition of exogenous cellulases and xylanases, CPB using *C. phytofermentans *can yield approximately 70% of the yield of SSF using engineered *S. cerevisiae *[28].

In this paper, we report on an assay that provides the ability to measure the influence of variation in biomass composition, pre-treatment methods, and conversion processes on digestibility, and thereby determine the potential effects of numerous variables in biofuel production. In addition to sorghum, feedstock quality was evaluated for cultivars of shrub willow (*Salix *spp.) and *Brachypodium distachyon *accessions to demonstrate the applicability of this assay for a wide range of feedstocks, from woody crops to herbaceous grasses. The *C. phytofermentans *bioassay provides a direct and quantitative means of assessing feedstock quality, both in terms of digestibility and conversion.

## Results

### Effect of feedstock concentration and time on ethanol concentration

**Figure 1:**
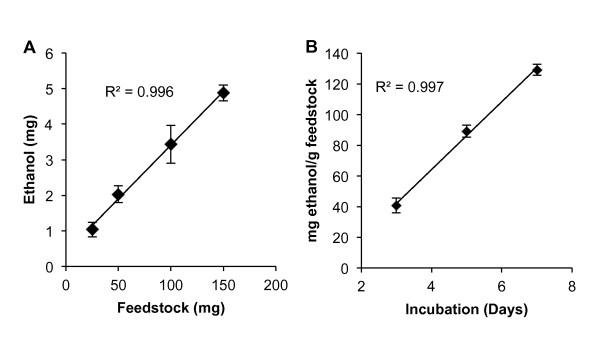
**Ethanol concentration increased linearly with time and feedstock concentration**. Effect of feedstock concentration **(A) **and time **(B) **on ethanol yield (mean ± SEM) by *Clostridium phytofermentans *growing on wild-type *Sorghum bicolor *(three to five experiments).

We evaluated the effect of *S. bicolor *wild-type biomass concentrations ranging from 2.5 to 15.0 g/l on ethanol concentration in supernatant after 3 days of incubation with *C. phytofermentans*. The concentration of ethanol increased by 0.03 mg ethanol/mg feedstock, and the replicated treatments were a good fit to a linear model (Figure 1A). All subsequent experiments were conducted using 5.0 g/l (or 50 mg in 10 ml) of liquid media. To determine the effect of time after inoculation on ethanol yield, samples were incubated for 3, 5, and 7 days. Similar to the trend observed with the increased feedstock concentration, ethanol production increased linearly, by 22.13 mg ethanol/g feedstock per day (Figure 1B). Thus, to facilitate rapid assays, the shortest incubation period of 3 days was used for all subsequent experiments.

### Effect of lignin mutants on *Clostridium phytofermentans *polysaccharide metabolism and ethanol concentration

**Figure 2:**
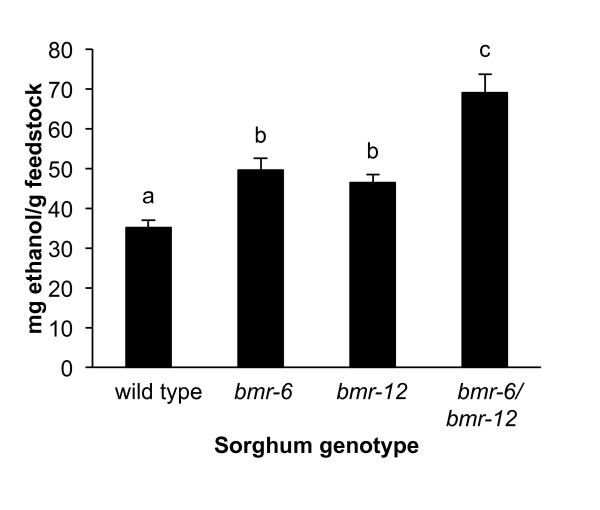
**Sorghum *brown midrib *lignin mutants yield more ethanol than wild type**. Degrained field-grown plants were ground and incubated for 3 days with *Clostridium phytofermentans*. Data are mean ± SEM of three separate experiments. Bars annotated with the same letter are not significantly different at *P *< 0.05 (Student's *t*-test).

We tested wild-type *Sorghum bicolor *and three well-characterized *brown midrib *(*bmr*) lignin mutants of sorghum (*bmr-6*, *bmr-12*, and a double mutant *bmr-6*/*bmr-12*) with the established assay conditions. A significant and nearly two-fold range of ethanol concentration was seen (Figure 2). The wild-type sorghum yielded approximately 62 mg ethanol/g feedstock. The single mutants *bmr-6 *and *bmr-12*, which have loss-of-function mutations in the *CAD *and caffeic acid *O*-methyltransferase (*COMT*) genes, respectively, yielded approximately 33% more ethanol than the wild-type plant. Although not significantly different across three experiments, *bmr-6 *consistently yielded slightly more ethanol than *bmr-12*. Exhibiting a somewhat additive effect, the *bmr-6*/*bmr-12 *mutant harboring both mutations in the wild-type background yielded an average of 113 mg ethanol/g feedstock.

**Figure 3:**
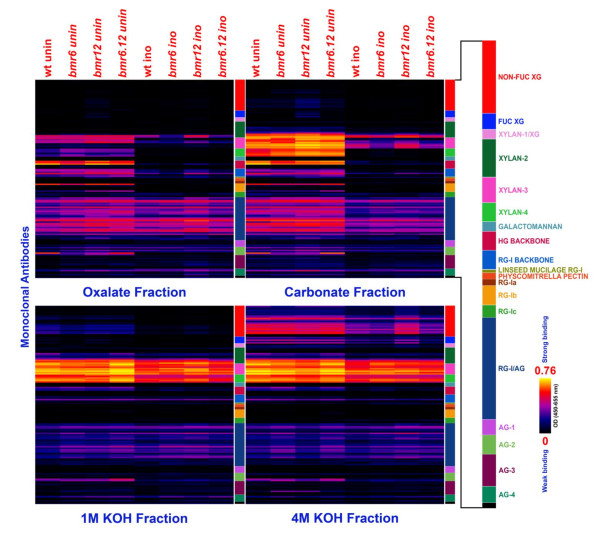
**Glycome profiling of *Sorghum bicolor *near-isogenic lines before and after inoculation with *Clostridium phytofermentans***. Sequential extractions using **(A) **oxalate, **(B) **carbonate, and (**C) **1 mol/l and **(D) **4 mol/l potassium hydroxide of five replicates of inoculated and uninoculated wild type, *bmr-6*, *bmr-12*, and *bmr-6/bmr-12 *sorghum were generated as described previously [29]. ELISAs using 147 monoclonal antibodies directed against glycan were used to detect the presence of cell-wall glycan epitopes in each extraction fraction, and the data are presented as heat maps (see Additional file 1; see Additional file 2). Plant biomass samples tested are indicated at the top of each column. Antibodies are grouped according to the recognized glycan are listed on the right panel. The black-red-white scale indicates the strength of the ELISA signal: bright-red, white, and dark-blue colors depict strong, medium, and no binding, respectively. AG, arabinogalactan; FUC-XG, fucosylated xyloglucan; HG, homogalacturonan; RG, rhamnogalacturonan.

To better understand feedstock utilization by the microbe across the different sorghum genotypes, we conducted glycome profiling of all four *S. bicolor *genotypes before and after inoculation with *C. phytofermentans*, using 147 glycan-directed monoclonal antibodies (see Additional file 1) [29]. With this approach, we were able to measure the differences in the abundance of extractable polysaccharide epitopes between genotypes, and the effect of microbial inoculation (Figure 3; see Additional file 2). Significant reductions in the abundance of oxalate and carbonate-extractable cell-wall polysaccharide epitopes were noted between inoculated and uninoculated samples. There was a substantial removal of xylan epitopes recognized by the xylan-2, -3 and -4 groups of antibodies, and of pectic backbone epitopes recognized by the homogalacturonan backbone and rhamnogalacturonan-I backbone groups of antibodies from the oxalate extracts of *C. phytofermentans*-treated samples. The *bmr *mutants contained more of the oxalate-extractable xylan-4 epitopes than did the wild-type plant tissue, with *bmr-6 *showing the largest increase. Inoculation with *C. phytofermentans *resulted in removal of many of the oxalate and carbonate-extractable xylan-3 epitopes, although some xylan-3 epitopes appeared to be more resistant to degradation than others (Figure 3). The oxalate-extractable xylan-3 epitopes were substantially removed from the *bmr-6 *and *bmr-6/bmr-12 *walls after inoculation. The effect of bacterial inoculation was also dramatic in the carbonate-extractable xylan-3 epitopes from *bmr-6 *and *bmr6-bmr-12 *walls. In carbonate extracts of inoculated samples, a significant reduction in the abundance of pectic-arabinogalactan epitopes recognized by the rhamnogalacturonan-I/arabinogalactan group of antibodies was also apparent. Xyloglucan epitopes extracted by 4 mol/l potassium hydroxide (KOH) were significantly reduced in microbe-treated wild type, *bmr-6 *and *bmr-6*/*bmr-12 *plants, and only subtle reductions in various xylan epitopes were noted in the 1 mol/l and 4 mol/l KOH extracts. Lastly, a marked reduction in arabinogalactan epitopes recognized by the arabinogalactan-2, -3, and -4 antibodies was seen in all wall extracts from inoculated samples.

**Figure 4:**
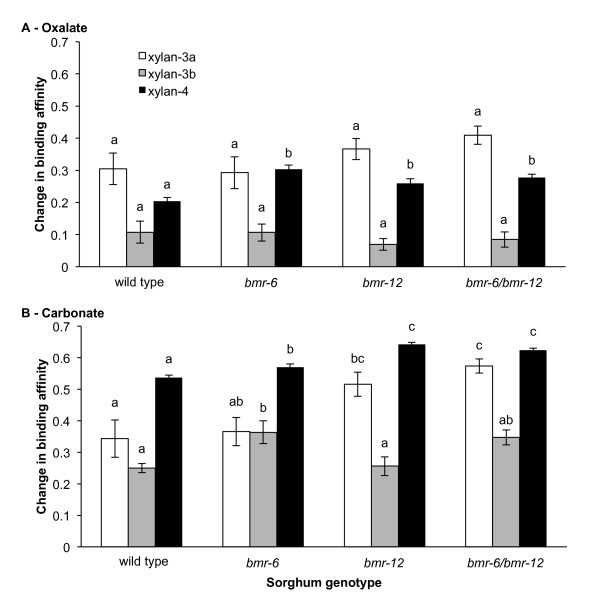
**Polysaccharide conversion by *Clostridium pytofermentans *of wild type and *brown midrib *mutant sorghum**. The remaining biomass pellet was given sequential extractions using **(A) **oxalate, **(B) **carbonate, and 1 mol/l and 4 mol/l potassium hydroxide followed by glycome profiling [29]. For each genotype, values represent the difference in extractable xylan between the mean ± SEM of five uninoculated samples and each of the inoculated samples. Xylan-3a (CCRCM-137, -149, -160), -3b (CCRCM-144, -146, -152, -155), and -4 (CCRCM-138, -139, -140, -148, -151, -153) are groups of antibodies generated using either oat or corncob xylan as an immunogen, and are classified by hierarchical clustering analysis of their recognition patterns. Data are mean ± SEM of five replicates; bars annotated with the same letter are not significantly different at *P *< 0.05 (Scheffé's test for multiple comparisons).

The grass family Poaceae, which includes *S. bicolor*, has a typical stem composition consisting of cellulose, lignin, and the hemicellulose arabinoxylan [30]. Therefore, we focused our attention on the relative abundance of xylan epitopes as detected by 14 antibodies that recognize a wide range of xylan epitopes [29]. To further examine the effect of bacterial inoculation, we the assessed the difference between each of the five inoculated replicates, and calculated the mean of the uninoculated ELISA values for each genotype. These values are an estimate of polysaccharide conversion by *C. phytofermentans*. The antibody groups xylan-1 and xylan-2 tended not to change between treatment or genotype, or did not bind to the wall at all. Even though xylans were present in the 1 mol/l and 4 mol/l KOH-extractable fractions, only very small changes were seen after inoculation, regardless of genotype. However, within the oxalate-extractable fraction, the xylan-4 antibodies detected a significantly smaller change in wild-type xylan compared with the *bmr-6 *mutant xylan (Figure 4A). In the carbonate-extractable fraction, there was a smaller effect of inoculation, as detected by the xylan-3b antibodies, on the wild-type plant than on the *bmr-6 *and *bmr-6*/*bmr-12 *mutants (Figure 4B), and a smaller effect of inoculation, as detected by the xylan-4 antibodies, on the wild-type plant than on the *bmr-12 *and *bmr-6*/*bmr-12 *mutants (Figure 4B). Overall, the changes in extractable polysaccharide epitopes of the cell-wall fractions indicate that loosely integrated xylans and pectins are the primary polysaccharide targets of *C. phytofermentans*, and that these are more accessible in the *bmr *mutants than in the wild type.

### Effect of feedstock particle size on ethanol concentration

Among the many pleiotropic effects of *bmr *mutations in sorghum is an effect on the rigidity of cell walls, reflected as increased lodging of field-grown plants [31]. That being the case, it might be possible that differences in digestibility, measured by ethanol concentration, are a product of differences between genotypes in the particle-size distribution after grinding.

**Figure 5:**
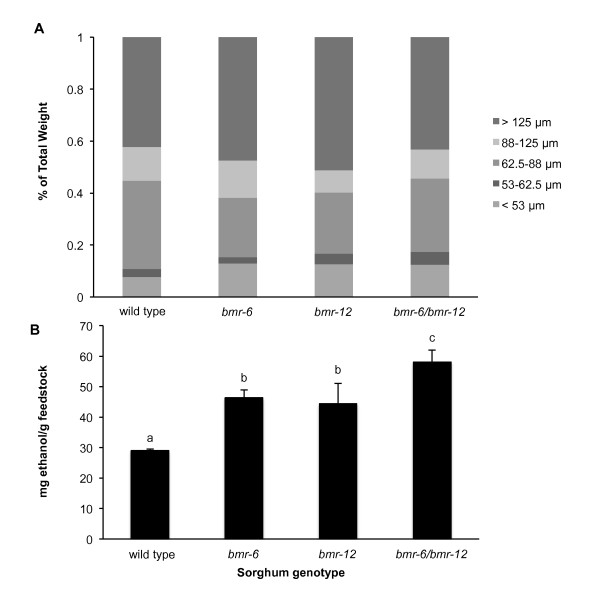
**Genetic differences in ethanol yield are not the result of differential particle size of sorghum near-isogenic lines**. **(A) **After grinding, replicate samples (*n *= 3) were subject to analytical sieving, and fractions weighed to determine the percentage of total weight. **(B) **The 53 to 62.5 μm sieve fraction was tested for ethanol yield as previously described. Means ± SEM annotated with the same letter are not significantly different at *P *< 0.05 (Student's *t*-test).

In this study, ground and washed plant material was separated based on particle size by analytical sieving before inoculation (Figure 5A). The largest fraction was greater than 125 μm. and approximately 10% of the samples were composed of particles smaller than 53 μm. The fraction ranging from 53 to 62.5 μm was tested for digestibility, and the results were similar to two experiments in which all fractions were tested (Figure 5B). The wild-type plant yielded significantly less ethanol than the *bmr6 *and *bmr12 *mutants. The double mutant once again exhibited an additive effect, with nearly twice the ethanol concentration as the wild type. Again, the single mutants were not significantly different from each other. Thus, when the particle size of the plant feedstock was held within a very narrow range, results were similar to those from experiments using the complete range of particle sizes.

### The effect of genetic variation on the biological conversion efficiency of sorghum landraces, *Brachypodium distachyon *accessions, and shrub-willow germplasm

We measured feedstock quality of 16 sorghum landrace populations originating from western, north-central, eastern, and southern regions of Africa. together with a modern cultivar developed in the USA, RTx430. The plants sampled were S_1 _families, that is, the progeny of a single self-fertilized individual from Plant Introductions (Plant Genetic Resources Conservation Unit, Griffin, GA, USA). The family means from the bioassay ranged from 26.5 to 39.4 mg ethanol/g feedstock (Table 1). Accessions PI 300120 and PI 365024 did not produce detectable quantities of ethanol, and were therefore not included in the analysis. The percentage of the variance contributed by each source of variation for ethanol yield was estimated by calculation of variance components (Table 2). Variance components attributed to genotype accounted for 72.49% of the variance, and there was no significant effect of *S. bicolor *subspecies or country of origin. Analysis of variance of three independent 'Atlas' near-isogenic line (NIL) experiments revealed that the effects of experiment and genotype were significant (*P <*0.01). There was no significant genotype × experiment interaction effect, therefore, no change in the magnitude of the differences between genotypes across experiments was seen. Variance components attributed to genotype were greater than those for environment and genotype × environment interaction.

**Table 1:** Mean values for supernatant ethanol concentration of S_1 _families derived from *Sorghum bicolor ssp*. Accessions 3d after inoculation with *Clostridium phytofermentans*.

Accession	***Sorghum *ssp**.	Origin	Ethanol, mg ethanol/g* feedstock^†^
PI 549183	*Sorghum bicolor*	Chad	39.4^a^

PI 208190	*Sorghum verticilliflorum*	South Africa	37.0^ab^

RTx430	*S. bicolor*	U.S.A.	35.0^bc^

PI 549194	*S. bicolor*	Chad	34.1^bc^

PI 549179	*S. bicolor*	Mauritania	34.0^bc^

PI 226096	*S. verticilliflorum*	Kenya	32.2^cd^

PI 329252	*S. verticilliflorum*	Ethiopia	30.6^de^

PI 302111	*S. verticilliflorum*	South Africa	30.2^de^

PI 521353	*Sorghum drummondii*	Kenya	30.1^de^

PI 549202	*S. bicolor*	Chad	30.0^def^

PI 302113	*S. verticilliflorum*	South Africa	30.0^def^

PI 521355	*S. drummondii*	Kenya	29.3^def^

PI 156549	*S. verticilliflorum*	Zimbabwe	28.6^ef^

PI 300117	*S. verticilliflorum*	South Africa	28.6^ef^

PI 520775	*S. drummondii*	Kenya	26.5^f^

PI 300120	*S. verticilliflorum*	South Africa	No data

PI 365024	*S. verticilliflorum*	South Africa	No data

*Values followed by the same letter are not significantly different at *P <*0.05 based on Scheffé's test for multiple comparisons.

**Table 2:** Genetic effects account for most of the variation measured by the feedstock quality assay.

Source of variation	Accessions tested, %^a^	
	
	Near-isogenic lines	Landraces
Experiment	7.50*	NA^b^
Replication	3.52	1.32
Genotype	64.12*	72.49*
Genotype × experiment	2.32	NA
Error	22.54	26.19

^a^Percentage of the total variance for each source of variation for supernatant ethanol concentration of experiment 1 (*S. bicolor bmr *near-isogenic lines) and experiment 2 (15 *S. bicolor ssp*. accessions) 3 days after inoculation with ***Clostridium ****phytofermentans*.

^b^NA, not applicable.

*Significant at the 0.01 probability level.

A similar continuous range in ethanol concentration was seen within a collection of 14 shrub-willow genotypes (Table 3). The genotype means showed no pattern with parentage of the seven *Salix *species pedigrees. The cultivar with the highest ethanol yield, 'Canastota', produced a mean concentration of 38.3 mg ethanol/g feedstock. The cultivar with the lowest feedstock quality, 'Otisco', produced 26.9 mg ethanol/g feedstock.

**Table 3:** Mean values for supernatant ethanol concentration of shrub willow (*Salix *spp.) cultivars 5d after inoculation with *Clostridium phytofermentans*.

Cultivar name	Pedigree	Ethanol, mg ethanol/g feedstock*
Canastota	*S. sacchalinensis*/*S. miyabeana*	38.3^a^
Fabius	*S. viminalis*/*S. miyabeana*	35.4^ab^
Onondaga	*S. purpurea*	35.0^ab^
Preble	*S. viminalis*/*S. sacchalinensis*/*S. miyabeana*	34.5^ab^
Sherburne	*S. sacchalinensis*/*S. miyabeana*	34.1^abc^
94006	*S. purpurea*	33.2^bcd^
Fish Creek	*S. purpurea*	33.0^bcd^
Oneida	*S. purpurea*/*S. miyabeana*	31.7^bcde^
SV1	*S. viminalis *hybrid	31.3^bcdef^
Allegany	*S. koriyangi */*S. viminalis*	29.8^cdef^
94001	*S. purpurea*	29.4^def^
Millbrook	*S. purpurea*/*S. miyabeana*	29.0^def^
Owasco	*S. viminalis*/*S. miyabeana*	27.9^ef^
Otisco	*S. viminalis*/*S. miyabeana*	26.9^f^
		

*Values followed by the same letter are not significantly different at *P <*0.05 based on Scheffé's test for multiple comparisons.

**Figure 6:**
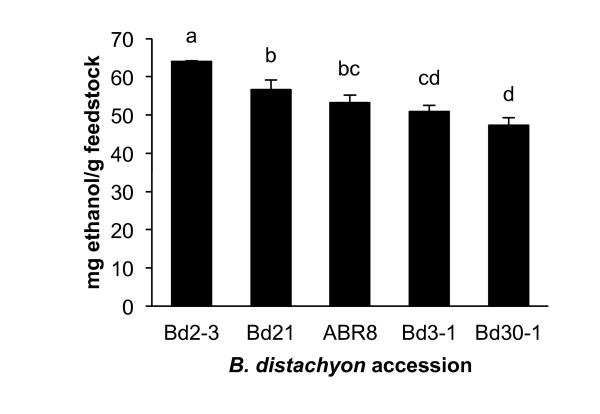
**Natural variation in ethanol yield among five *Brachypodium distachyon *accessions**. Stems of fully senesced plants were ground and incubated for 3 days with *Clostridium phytofermentans*. Data are means ± SEM of three replicates; bars annotated with the same letter are not significantly different at *P *< 0.05 (Scheffé's test for multiple comparisons).

Five accessions of the energy-crop model species *B. distachyon *exhibited a supernatant ethanol range of 47.4 to 64.0 mg ethanol/g feedstock. Scheffé's multiple range test separated the accessions into distinct groups (Figure 6). Two genetically similar accessions from Iraq, Bd2-3 and Bd3-1, were the most and second least digestible accessions, respectively. The accession yielding the lowest supernatant ethanol concentration (47.35 mg ethanol/g feedstock) was Bd30-1, which originated from Spain. The average supernatant ethanol concentrations of the sorghum landraces (31.7 mg ethanol/g feedstock) and the willow cultivars (32.1 mg ethanol/g feedstock after 5 days incubation) were considerably lower than that of the *B. distachyon *accessions (54.4 mg ethanol/g feedstock).

## Discussion

Some key crop species, such as maize, wheat, and rice, have been domesticated from about 10,000 years ago, and have been under continuous selection for important agronomic and quality traits. Whereas progenitor species of many crops are unrecognizable from their domesticated counterparts, many of our potential energy crops are at best just a few crosses away from the wild accessions. Miscanthus, switchgrass, energy cane, and willow are just beginning the process of domestication. Thus, there is equivalent potential for improvement of fuel yield per unit of land through breeding of energy crops, but this must be accomplished in an abbreviated timeframe to have an effect on global climate change [32,33]. One trait to improve upon is the ease of biomass deconstruction to simple sugars that are suitable for conversion to a liquid fuel [34]. Many species have demonstrated genetic variation in digestibility for both forage quality and enzymatic digestion, thus traditional plant breeding facilitated by suitable phenotypic assays, such as the bioconversion approach described here, will lead to a more efficient biofuels industry [3,35]. The overall content and composition of cell-wall lignin has proven to be a significant determining factor for lignocellulosic biomass digestibility [7,8]. In the 1920s, agronomists identified the first lignin mutants in maize as genotypes with characteristic browning of the leaf midrib, hence the mutant name '*brown midrib*' [36]. Negative perturbation of multiple points in the lignin biosynthetic pathway results in increased digestibility in numerous species including maize, sorghum, switchgrass, tall fescue, alfalfa, tobacco, and poplar [4,7]. In both sorghum and maize, mutations in the *CAD *or *COMT *genes have been identified as causes for the *brown midrib *phenotype [37-39].

The bioconversion assay described here was developed using a well-characterized set of sorghum *bmr *NILs, the same samples previously described by Dien. [23]. In this study, the Klason lignin content was 15% lower in the *bmr-6 *and *bmr-12 *single mutants than in wild-type sorghum, and nearly twice as low (27%) in the *bmr-6*/*bmr-12 *double mutant. Interestingly, no other differences were between the NILs were seen for carbohydrate content or non-lignin-associated measures of *in vitro *digestibility, such as neutral or acid detergent fiber [23]. Therefore, the differential effects of *C. phytofermentans *inoculation on NILs is not due to the total amount of digestible sugars in the NILs, but rather the accessibility of those polysaccharides to enzymatic digestion, which is therefore strongly influenced by lignin content. Fermentation of the same material by *S. cerevisiae *after dilute acid pre-treatment and cellulase and β-glucosidase enzymatic digestion produced improvements in ethanol-conversion efficiency of 11% and 17% for the single and double mutants, respectively [23]. The differences in ethanol production by NILs digested by *S. cerevisiae *were far less pronounced than those digested with *C. phytofermentans*. Considering that xylose yield was roughly constant across the NILs, and that *S. cerevisiae *is capable of fermenting hexoses only, it is likely that the differences in ethanol yield were due to differential enzymatic release of glucose. *C. phytofermentans *digests plant biomass with secreted cellulolytic enzymes, and subsequently imports and metabolizes pentoses and hexoses. Thus, ethanol yield is a combined measure of the digestibility of hemicelluloses and cellulose.

To gain a greater understanding into why ethanol yields might be greater with the *bmr *mutants, we assayed the polysaccharide content both before and after inoculation, using a comprehensive collection of 147 glycan-directed monoclonal antibodies to quantify changes in most major classes of cell-wall polysaccharides [29]. Considering the minute quantities of pectins in sorghum stems [40], we focused on changes in the abundance of xylan, which accounted for a substantial proportion of the overall biomass content [23]. The relative amount of extractable xylan differed between the NILs, as did the difference in extractable xylan metabolized by *C. phytofermentans*. Therefore, the increased quantities of ethanol produced from the *bmr *mutants is in part due to the increased accessibility of xylan to hydrolysis.

Although it was apparent that there are genetic differences in plant properties that influence feedstock digestibility, several analyses of sorghum, maize, and alfalfa lignin mutants revealed genotype × chemical pre-treatment interaction effects. In other words, the magnitude of the differences between genotypes or even their rank order will change depending on the method of pre-treatment. Enzymatic release of glucose from the maize lignin mutant *bm1 *was equivalent to that of wild type after alkali pre-treatment, but significantly greater after acid pre-treatment [19]. Relative to untreated sorghum, the difference between wild-type sorghum and the sorghum lignin mutants *bmr-3 *and *bmr-12 *diminished after dilute acid pre-treatment [41]. In the same study, mutations in other loci resulting in a *bmr *phenotype produced little or no change in response to pre-treatment. Similarly, the magnitude of the difference in enzymatic saccharification between wild-type alfalfa and plants downregulated for several different lignin genes varied in response to acid pre-treatment [8]. In the case of plants downregulated for *CCoAMT*, a significant difference from wild type was seen only in the absence of pre-treatment. By contrast, the difference in hydrolysis efficiency between *C4H *and *HCT *downregulated plants and wild type was twice as large in the absence of pre-treatment. After treatment with 15% ammonium hydroxide the hydrolysis yield potential of a large panel of sorghum accessions ranged from the same level as that of the untreated sorghum up to an approximately threefold increase over the untreated plants [42]. The goal of pre-treatment is to improve the accessibility of the polysaccharides to hydrolysis [43]. Clearly, there is a complex relationship between efficiency of feedstock utilization and pre-treatment. In this study, we attempted to maximize the ease of conducting the bioconversion quality assay and to maximize the sensitivity of the assay to detect genetic differences. Although small differences in genetic potential may not be relevant in certain industrial scenarios, detection remains important for development of energy crops. Consistent and incremental progress in modern plant breeding is the product of changing the frequencies of alleles with small additive effects. Thus, a sensitive assay is required to detect such variation, and certain pretreatments may diminish sensitivity.

We measured significant genetic variation among collections of two plant taxa considered to have excellent potential as energy crops, sorghum and shrub willow, and a model for grasses, *B. distachyon *[44-46]. The variation was notable considering that the samples tested by no means represent the overall genetic variation within each taxon. Thus, we would expect a wider range of phenotypic variation with an increase in sample number and diversity. The ethanol yields from sorghum were similar to those from willow, ranging from 26.5 to 39.4 mg ethanol/g feedstock. For willow, no significant correlations were seen between ethanol yield and biomass compositional data (Serapiglia and Smart, unpublished data). Most of the variation could be attributed to genetics rather than to experimental differences; consequently, routine gain from selection could be expected in the range of variation detected. At the same time, the assay could clearly identify differences between major genetic perturbations in biomass composition (typical of the *bmr *mutations) and continuous variation among natural and segregating populations. The most digestible genotypes from all three biofuel crop taxa yielded approximately 25% more ethanol that the most recalcitrant. This is only slightly less than the differences between wild-type sorghum and the single-gene *bmr *mutants. The quantity of material tested is well within the amount of stem biomass typically produced by a single plant of rapid-cycling model plants such as *Arabidopsis thaliana *and *B. distachyon*. We are currently using this system in a 96-well format, which, combined with a commercial robotics preparation [19,22], is capable of throughput levels required by a core feedstock testing facility for plant breeding and mutant screens.

## Conclusions

In this paper we have described a laboratory assay that quantifies the amount of ethanol produced by *C. phytofermentans *cultured on plant biomass. This approach may be useful to estimate the potential effects of pre-treatment, conversion methods, and microbial and plant genetic diversity on biofuel manufacturing. The assay is capable of measuring subtle genetic differences within energy crops and the research model species *B. distachyon*, making it particularly useful for genetic analysis of the mechanisms underlying plant feedstock amenability to biological conversion. The *C. phytofermentans *bioassay provides a means of assessing feedstock quality in terms of both digestibility and conversion.

## Methods

### Plant material

Genetic material consisted of *Sorghum bicolor *'Atlas' (designated as the wild type for this analysis), and three near-isogenic *brown midrib *(*bmr*) mutant lines: *bmr-6*, *bmr-12*, and the double mutant *bmr-6/bmr-12 *[47]. Degrained plant samples were collected as previously described [23] from a single field at the University of Nebraska Agricultural Research and Development Station (Lincoln, NE, USA) in the summer of 2005. Briefly, when all plants reached the hard-dough stage of maturity, samples of all four genotypes were taken from each of two replicate plots arranged in randomized complete blocks. After harvesting by flail chopper, samples were oven-dried at 50°C. In a separate experiment, 17 accessions of *S. bicolor *subspecies *bicolor*, *Sorghum verticilliflorum*, and *Sorghum drummondii *were collected in the summer of 2010 (Table 1). All but one accession, cultivar RTx430 [48], are S_1 _families derived from Plant Introduction accessions obtained from the Plant Genetic Resources Conservation Unit, and represent an array of *S. bicolor *subspecies diversity. The replicates were composited to create a single sample for each genotype. All plant biomass samples were then washed using a hot ethanol solution to remove free sugars. First, the samples were placed in 50 ml plastic screw cap tubes, filled with 70% v/v ethanol solution, and then incubated at 70**°**C for 1 hour. After 30 minutes, the supernatant was discarded, replaced with fresh ethanol solution, and returned to the water bath for an additional 30 minutes. Samples were then given a final rinse with a 70% (v/v) methanol solution and subsequently air-dried for 24 hours in a fume hood. Using a 25.0 ml stainless-steel grinding jar (catalog number #02.462.0213; Retsch Inc, Newtown, PA) with one stainless-steel grinding ball of 15 mm in size (catalog number 05.368.0109; Retsch) per jar, samples were homogenized (Mixer Mill MM400; Retsch) for 3 minutes at 30 Hz. Particle-size determinations were made using the USP General Test 786 Method I by analytical sieving. First, 5 g of dry plant tissue was placed on the top of the sieves and the apparatus was covered. The ground plant tissue was then passed through increasingly fine wire-mesh sieves (US standard sieves #80 (177 μm), #120 (125 μm), #160 (88 μm), #230 (62 μm), #270 (53 μm)). The sieves were shaken vigorously for 1 minute, then each sieve was carefully weighed, covered again, and the process resumed until the weight of each fraction was within 5% of the previous measurement. At that time, the fractions in each sieve were carefully collected, and final measurements taken. The weight of each sieve fraction was recorded, and divided by the total weight to determine the percentage each fraction represented.

Dry seeds from five inbred *B. distachyon *accessions were sown directly into 100 mm pots containing potting mix (#2; Conrad Fafard Inc., Agawa, MA, USA) Growth chamber temperature was maintained at 20°C with a 20 hour light/4 hour dark cycle at a fluence rate of 200 μmol of photons^/^m^2^/s, and relative humidity of 68.0 to 68.5. Fully senesced stems were washed and milled as described above. Seed of Bd30-1, Bd3-1, Bd21, and Bd2-3 were kindly provided by David F. Garvin (USDA-ARS) and seed of ABR8 by John Draper (Aberystwyth University, UK).

Shrub willow (*Salix *spp.) biomass samples from 14 genetically diverse genotypes were selected for conversion-efficiency analysis. These included commercial cultivars selected from controlled intraspecies and interspecies crosses, and unimproved accessions obtained from naturally established stands in northeastern USA and Canada. Biomass was harvested in December 2009 after the third post-coppice season from plants growing in four replicate plots in the 2006 Yield Trial in Constableville, NY, USA. Stems were chipped. and the four replicates pooled. The chips were dried to a constant weight at 65°C and rough-milled using a mill (Wiley mill; Thomas Scientific, Swedesboro, NJ, USA) with a 20-mesh screen. Further fine milling down to a particle size of 0.5 μm was performed using an analytical mill (MF 10; IKA, Willmington, NC, USA). Samples were then milled and washed as described above for sorghum.

### Ethanol analysis

*C. phytofermentans *ISDg (ATCC 700394) was cultured in a defined medium, MQM5.1 (2.0 g/l Na H_2_PO_4_, 10.0 g/l K_2_HPO_4_, 1.0 g/l (NH_4_)_2_SO_4_, 1.0 g/l L-cysteine hydrochloride monohydrate, 20 ml/l XT solution (5.0 g/l xanthine and 5.0 g/l thymine in 0.06 N NaOH) 10 ml/l AA1 solution (.0 g/l of each of the following amino acids: alanine, arginine, histidine, isoleucine, leucine, methinonine, proline and valine), and 10 ml/l Bach's trace element (BTE) solution [49]), Resazurin (1 mg/l) was added as an oxidation/reduction indicator. After autoclaving, 10 ml/l CPV3 solution (20 mg/l *p*-aminobenzoic acid, 1 mg/l biotin, 30 mg/l folinic acid, 80 mg/l nicotinamide, 5 mg/l pantethine, 2 mg/l pyridoxal hydrochloride, 30 mg/l riboflavin, and 10 mg/l thiamine) was added.

*The C. phytofermentans *inoculum was initially grown in MQM5.1 with 3 g/l cellobiose using the anaerobic techniques described by Hungate [50] in 10 ml volumes in 18 × 180 mm tubes sealed with neoprene stoppers.

For the biological conversion- quality assay, Hungate tubes, containing 50 mg of plant tissue and 10 ml of MQM5.1 media were inoculated with 0.1 ml of the initial culture. Sorghum and *B. distachyon *samples were incubated without shaking for 72 hours at 37°C, unless specified otherwise. Willow samples were incubated for 120 hours. At that time, 1.0 ml of each sample supernatant was collected and filtered using a 0.22 μm syringe-driven filter unit (Millipore Corp., Billerica, MA, USA). Samples were separated using an HPLC (Shimadzu Corp., Kyoto, Japan) equipped with a carbohydrate analysis column (300 mm × 7.8 mm; Aminex HPX-87H; Bio-Rad Laboratories, Inc., Hercules, CA, USA) and a refractive-index detector. The column was operated at 30°C with 0.005 N sulfuric acid as the running buffer at a rate of 0.7 ml/min, and 5.0 μL sample injections. Retention time for ethanol (17.84 ± 0.02 minutes), was determined using a commercial mix (Fuel Ethanol Residual Saccharides Mix; catalog number 48468-U; Sigma-Aldrich, St Louis, MO, USA) containing glycerol, glucose, maltotriose, maltose monohydrate, lactic acid, acetic acid, dextrin, and ethanol. Standards were run at the beginning, middle, and end of every distinct HPLC analysis to ensure accuracy and precision of measurements.

### Cell-wall extraction and glycome profiling

Approximately 100 mg of wild type and *bmr *mutant sorghum biomass, either inoculated with *C. phytofermentans *or uninoculated, were washed sequentially with absolute ethanol and acetone. The biomass materials were dried overnight in a fume hood at room temperature. The dry residues were sequentially extracted with increasingly harsh reagents at suspensions of 10 mg/ml (based on starting biomass weight used) to obtain fractions enriched with cell-wall components. The biomass was first incubated as a suspension in 50 mmol/l ammonium oxalate (pH 5.0) and mixed overnight at room temperature on a shaker. The suspension was then separated by centrifugation at 3400 *g *for 15 minutes at room temperature. The clear supernatant obtained was decanted, and saved as the ammonium oxalate fraction. The pellet was washed by suspending in the same volume of deionized water, followed by centrifugation as above. The resulting supernatant in the washing step was discarded. The pellet was then subjected to additional sequential extractions using in turn 50 mmol/l sodium carbonate (pH 10) with 0.5% sodium borohydride w/v, 1 mol/l containing 1% sodium borohydride w/v, and 4 mol/l KOH containing 1% sodium borohydride w/v following the same steps as described above to obtain the sodium carbonate, 1 mol/l KOH and 4 mol/l KOH fractions respectively. Both KOH fractions were neutralized using glacial acetic acid. All fractions were dialyzed using molecular-porous membrane tubing (Spectra/Por; Spectrum Laboratories Inc., Rancho Dominguez, CA, USA) with a nominal molecular-weight cut-off (MWCO) of 3,500 against four changes of deionized water (sample:water approximately 1:60) at room temperature for a total of 48 hours, and then lyophilized.

All extracts were first dissolved in deionized water to a concentration of 0.2 mg/ml. Total sugar content of the cell-wall extracts were then determined using the phenol-sulfuric acid method [51,52]. The extracts were subsequently diluted to the same sugar concentration of 60 μg sugar/ml for loading onto ELISA plates (Costar 3598; Corning Costar Corp., Corning, NY, USA). A sample (50 μL) of each diluted extract was added to each well and allowed to evaporate overnight at 37°C until dry. ELISA was conducted using an array of 147 monoclonal antibodies (see Additional file 1) specific to epitopes from most major groups of plant cell-wall polysaccharides as described previously [29]. ELISA data are presented as heat maps in which antibodies are grouped based on a hierarchical clustering analysis of their binding specificities against a diverse set of plant glycans [29].

### Statistical analysis

Three or four independent fermentation reactions were sampled at each time for each feedstock sample. Analysis of variance and Scheffé's test were performed using the agricolae package, and Student's *t*-test using a Bonferroni's correction for multiple comparisons in R v2.11.0. Significance was set a *P *< 0.05 or rP < 0.01.

## List of abbreviations

AA1: amino acid solution; BTE: Bach's trace element; CBP consolidated bioprocessing; KOH: potassium hydroxide; NIL: near-isogenic line; SSF: saccharification and fermentation; XT: xanthine-thymine.

## Competing interests

TAW and SBL have equity interest in Qteros, Inc.

## Authors' contributions

The experiments were conceived and designed by SJL, TAW, SP, JGAM, NFY, DJS, SBL, and SPH, and performed by SJL, SP, JGAM, HM, VB, and JFP. JFP, MGH, MJS and LBS contributed new reagents/analytical methods. SPH analyzed the data, and SJL, TAW, SP, MGH, MJS, and SPH wrote the paper. All authors read and approved the final manuscript.

## Supplementary Material

Additional file 1 **Schematic with hyperlinks to detailed descriptions of each antibody in the glycome platform**. Click here for file

Additional file 2 **Raw ELISA binding of data from four cell-wall extraction using 147 glycan-directed antibodies of *Sorghum bicolor *near-isogenic lines**. Click here for file
