# Supplementary material for: Biological conversion assay using Clostridium phytofermentans to estimate plant feedstock quality
Source: Biotechnol Biofuels. 2012 Feb 8;5:5. doi: 10.1186/1754-6834-5-5 (PMC3348094; doi:10.1186/1754-6834-5-5)
Supplement: Additional file 1 — Schematic with hyperlinks to detailed descriptions of each antibody in the glycome platform. [file 1754-6834-5-5-S1.PDF]

Non-Fucosylated Xyloglucan

CCRC-M54
CCRC-M48
CCRC-M49
CCRC-M96
CCRC-M50
CCRC-M51
CCRC-M53
CCRC-M100
CCRC-M103
CCRC-M58
CCRC-M86
CCRC-M55
CCRC-M52
CCRC-M99
CCRC-M95
CCRC-M101
CCRC-M104
CCRC-M89
CCRC-M93
CCRC-M87
CCRC-M88
CCRC-M57
CCRC-M90

Fucosylated Xyloglucan

CCRC-M102
CCRC-M39
CCRC-M106
CCRC-M84
CCRC-M1

Xylan 1/XG

CCRC-M111
CCRC-M108
CCRC-M109

Xylan 2

CCRC-M119
CCRC-M115
CCRC-M110
CCRC-M105
CCRC-M117
CCRC-M113
CCRC-M120
CCRC-M118
CCRC-M116

	CCRC-M114<br>CCRC-M154<br>CCRC-M150
Xylan 3	CCRC-M160<br>CCRC-M137<br>CCRC-M152<br>CCRC-M149<br>CCRC-M144<br>CCRC-M146<br>CCRC-M145<br>CCRC-M155
Xylan 4	CCRC-M153<br>CCRC-M151<br>CCRC-M148<br>CCRC-M140<br>CCRC-M139<br>CCRC-M138
Seed Galactomannan	CCRC-M75<br>CCRC-M70<br>CCRC-M74
Homogalacturonan Backbone	CCRC-M131<br>CCRC-M38<br>JIM5<br>JIM136<br>JIM7<br>CCRC-M34
Rhamnogalacturonan I Backbone	CCRC-M69<br>CCRC-M35<br>CCRC-M36<br>CCRC-M14<br>CCRC-M129<br>CCRC-M72
Linseed Mucilage Rhamnogalacturonan I	CCRC-M40
Physcomitrella Pectin	CCRC-M98<br>CCRC-M94

Rhamnogalacturonan IA

- CCRC-M5
- CCRC-M2

Rhamnogalacturonan IB

- CCRC-M23
- CCRC-M17
- CCRC-M19
- CCRC-M18
- CCRC-M56
- CCRC-M16

Rhamnogalacturonan IC

- JIM137
- JIM101
- CCRC-M61
- CCRC-M30

Rhamnogalacturonan I Arabinogalactan

- CCRC-M60
- CCRC-M41
- CCRC-M80
- CCRC-M79
- CCRC-M44
- CCRC-M33
- CCRC-M32
- CCRC-M13
- CCRC-M42
- CCRC-M24
- CCRC-M12
- CCRC-M7
- CCRC-M77
- CCRC-M25
- CCRC-M9
- CCRC-M128
- CCRC-M126
- CCRC-M134
- CCRC-M125
- CCRC-M123
- CCRC-M122
- CCRC-M121
- CCRC-M112
- CCRC-M21
- JIM131
- CCRC-M22
- JIM132
- JIM1

	CCRC-M15<br>CCRC-M8<br>MH4.3E5<br>JIM16
Arabinogalactan 1	JIM93<br>JIM94<br>JIM11<br>MAC204<br>JIM20
Arabinogalactan 2	JIM14<br>MAC207<br>JIM19<br>JIM12<br>CCRC-M133<br>CCRC-M107
Arabinogalactan 3	JIM4<br>CCRC-M31<br>JIM17<br>CCRC-M26<br>JIM15<br>JIM8<br>CCRC-M85<br>CCRC-M81<br>MAC266<br>PN 16.4B4
Arabinogalactan 4	JIM133<br>JIM13<br>CCRC-M92<br>CCRC-M91<br>CCRC-M78
Unidentified	MAC265<br>CCRC-M97
